# Nephropathic Cystinosis: Symptoms, Treatment, and Perspectives of a Systemic Disease

**DOI:** 10.3389/fped.2018.00058

**Published:** 2018-03-14

**Authors:** Sören Bäumner, Lutz T. Weber

**Affiliations:** ^1^Pediatric Nephrology, Children’s and Adolescents’ Hospital, University Hospital Cologne, Cologne, Germany

**Keywords:** nephropathic cystinosis, cysteamine, renal Fanconi syndrome, *CTNS* gene, hematopoietic stem cell therapy

## Abstract

Cystinosis is a rare autosomal recessive lysosomal storage disorder caused by mutations in the *CTNS* gene. Main dysfunction is a defective clearance of cystine from lysosomes that leads to accumulation of cystine crystals in every tissue of the body. There are three different forms: infantile nephropathic cystinosis, which is the most common form, juvenile nephropatic, and non-nephropathic cystinosis. Mostly, first symptom in infantile nephropathic cystinosis is renal Fanconi syndrome that occurs within the first year of life. Another prominent symptom is photophobia due to corneal crystal deposition. Cystine depletion therapy with cysteamine delays end-stage renal failure but does not stop progression of the disease. A new cysteamine formulation with delayed-release simplifies the administration schedule but still does not cure cystinosis. Even long-term depletion treatment resulting in bypassing the defective lysosomal transporter cannot reverse Fanconi syndrome. A future perspective offering a curative therapy may be transplantation of *CTNS*-carrying stem cells that has successfully been performed in mice.

## Introduction

Nephropathic cystinosis is a rare autosomal recessive lysosomal storage disorder leading to end-stage renal disease and many extra-renal complications with crystal deposition in the conjunctiva and cornea being the most prominent. It is caused by mutations in the *CTNS* gene on chromosome 17p13, which encodes the lysosomal cystin transporter cystinosin (1). First descriptions date to the beginning of the twentieth century, when cystine crystals were found in liver and spleen of a toddler, who died from dehydration and failure to thrive (2). More insight gave studies in the 1980s, where a defective clearance of cystine from lysosomes could be demonstrated (3, 4). The encoding *CTNS* gene was found in 1998 (5). Since then more than 100 pathogenic mutations in the *CTNS* gene have been described (6).

Central column of treatment is a depletion therapy with cysteamine that has proven to slow down progression of renal failure and to prevent or slow down extra-renal manifestations, even though it is not a curative therapy.

There are three different forms of cystinosis, which differ in age at manifestation and severity of the symptoms: (i) infantile nephropathic cystinosis, which is the most common and severe form; (ii) juvenile nephropathic cystinosis, which is characterized by later onset of symptoms and slower progression; and (iii) non-nephropatic cystinosis, with a mainly ocular manifestation, also known as adult form. Each form shows different mutations in the *CTNS* gene (7). Cystinosis occurs approximately in 1–2 of 100,000 live births (6).

## Infantile Nephropathic Cystinosis

Neonates are clinically asymptomatic at birth with normal birth-weight and normal length, even though cystine accumulation already starts *in utero*. First symptoms occur within the first year of life, usually presenting as renal Fanconi syndrome, a dysfunction of the proximal tubule that leads to polydipsia, polyuria, dehydration, proximal renal tubular acidosis, urinary loss of electrolytes, and growth retardation. In the urine, glucosuria and aminoaciduria can be found. In the case of glucosuria and normal serum glucose levels, one should always think of renal glucosuria or Fanconi syndrome. Glucosuria is the only parameter to be detected by urine dipstick in the Fanconi tubulopathy. The high protein turnover in the proximal tubule may explain why Fanconi syndrome is the first symptom of cystinosis.

Without treatment end-stage, renal failure occurs at a median age of 10 years. About 95% of cystinosis patients suffer from this type (6, 8). Historically cystinosis accounts for 5% of childhood renal failure (9).

## Juvenile Nephropathic Cystinosis

Patients with the juvenile type of nephropathic cystinosis develop symptoms at an older age, often presenting with more unspecific symptoms than patients with infantile cystinosis like nephrotic syndrome or mild proximal tubulopathy, but not necessarily the complete picture of Fanconi syndrome. End-stage renal disease may occur. Most patients are diagnosed in the second decade of life, when onset of photophobia leads to ocular examination and cystine crystals in the cornea can be found. This form accounts for approximately 5% of all cases of cystinosis (7, 10).

## Non-Nephropathic Cystinosis

This type presents only with ocular symptoms, as deposits are limited to cornea and conjunctiva and is also known as the adult form of cystinosis. Of note, there might be a continuum between milder forms of cystinosis, since non-nephropathic and juvenile forms have been described within one family. Therefore, renal function of every patient with non-nephropathic cystinosis should be monitored closely (10, 11).

## Diagnosis

First clinical signs in patients with infantile nephropathic cystinosis are polyuria, polydipsia, and failure to thrive. These symptoms reflect renal Fanconi syndrome in combination with metabolic acidosis and loss of electrolytes, especially phosphate (1). Since cystinosis is the most common reason for renal Fanconi syndrome at this age, this differential diagnosis should always be considered. Less common reasons for secondary Fanconi syndrome may be Dent’s disease, Lowe’s syndrome, inherited fructose intolerance, galactosemia, or tyrosinemia (12). Corneal cystine deposits, which are pathognomonic in untreated cystinosis, can rarely be found in the first year of life but are visible in almost every untreated patient at the age of 16 months (13). Confirmation of the diagnosis is made by measurement of elevated cystine levels in white blood cells followed by genetic testing for mutations in the *CTNS* gene (14). For cystine levels see Table 1.

**Table 1:** Diagnosis of cystinosis by measuring cystine levels.

Normal	≤0.2 nmol hemicystine/mg protein
Nephropathic cystinosis	5.0–23.0 nmol hemicystine/mg protein
Non-nephropathic cystinosis	1.0–3.0 nmol hemicystine/mg protein
Heterozygous carrier	≤1.0 nmol hemicystine/mg protein
Target levels for treated nephropathic cystinosis	<0.5–1.0 nmol hemicystin/mg protein

## Genetics

Confirmation of the diagnosis can be made by genetic testing. The *CTNS* gene, which encodes for the lysosomal carrier cystinosin, is located on the short arm of chromosome 17 (p13) (5). The most frequent mutation in Northern Europe is a 57-kb deletion that accounts for approximately 75% of all cases of nephropathic cystinosis (7, 15). Up to now, more than 100 mutations are known in the *CTNS* gene (6, 14). Most mutations in the *CTNS* gene result in a total loss of transport activity of cystinosin. The phenotype is the infantile nephropathic cystinosis. Patients with the milder juvenile or non-nephropathic form show different mutations suggesting that there is a genotype–phenotype correlation (11, 16–18). Still the pathogenesis is not fully understood because no disease model explains the link between lysosomal cystine accumulation and renal Fanconi syndrome completely (19).

## Pathophysiology and Clinical Presentation

**Figure 1:**
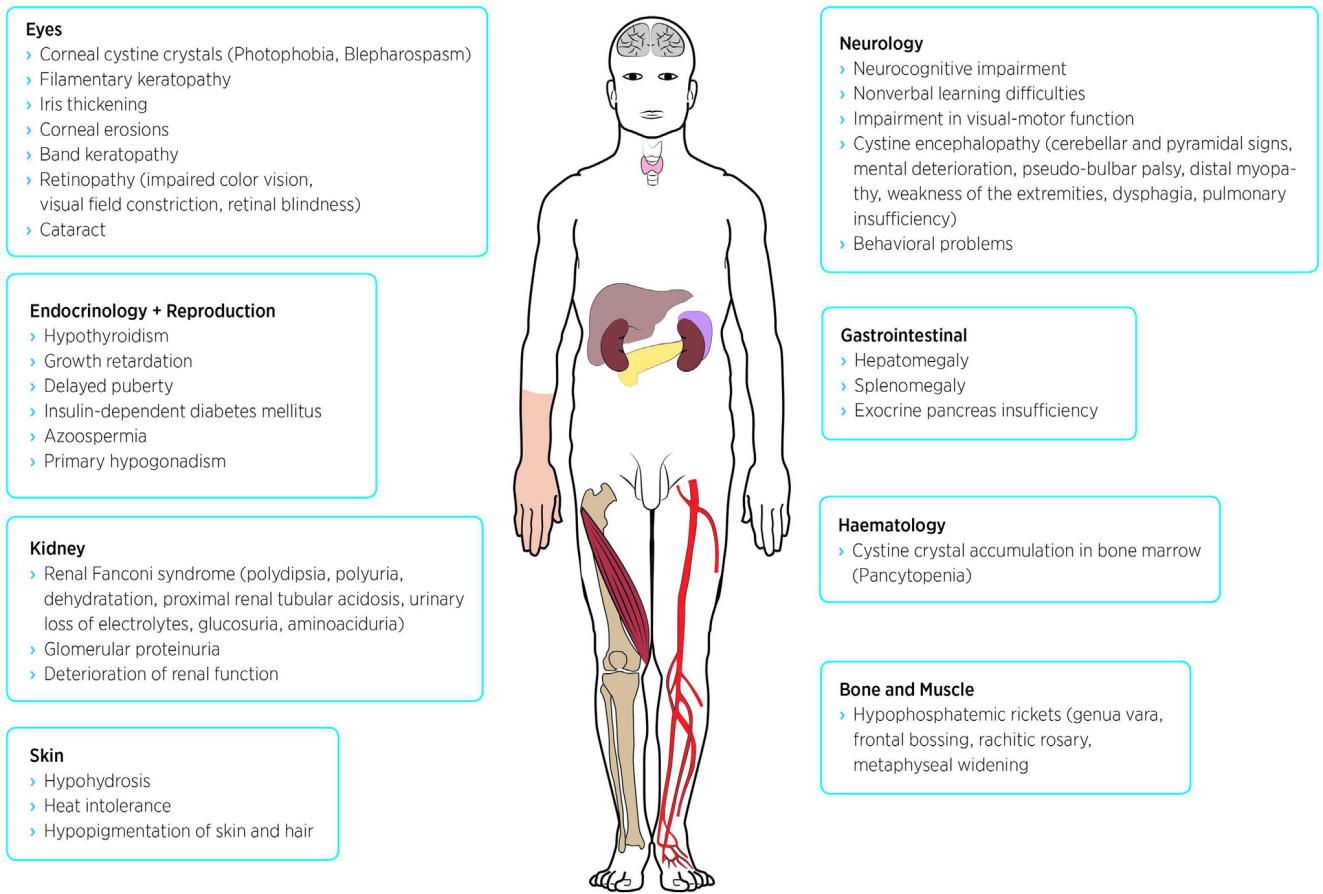
Possible symptoms and disease manifestations of nephropathic cystinosis.

Cystine is a disulfide of the amino acid cysteine. It is generated by lysosomal protein hydrolysis. Because no enzymatic dysfunction was found in cystinotic cells, research focused on defect transporter proteins leading to the identification of the seven-domain transmembrane protein cystinosin, which provides the transport of cystine from lysosomes to cytoplasm (20). Defective transport leads to accumulation and crystallization of cystine in the lysosomal compartment. Affected cells suffer from mitochondrial dysfunction, oxidative stress, and inflammation and, in the end, undergo apoptosis. Since lysosomes are part of every cell type cystine accumulation occurs throughout the whole body, making cystinosis a systemic disease (2). Over time, further symptoms can be seen in virtually every organ. The following sections represent just a selection of cystinosis manifestations. See also Figure 1.

## Kidney

Nonetheless, there is a different susceptibility of cell types to cystine accumulation with renal cells being especially susceptible. This is the reason why cystinosis presents primarily with renal symptoms (see above). Firstly impaired tissue is the proximal tubule leading to renal Fanconi syndrome, which is the major symptom of cystinosis. A characteristic histopathological sign is the so-called swan neck deformity, which describes the loss of proximal tubular cells. Electron microscopy shows cystine crystals in the tubular cells (10, 21, 22). Simultaneously, glomerular lesions occur due to involvement of podocytes that present histologically with the picture of focal and segmental sclerosis and lead clinically to glomerular proteinuria and progressive deterioration of renal function (2). Using electron microscopy, podocytes appear hypertrophic, multinucleate and have foot process effacement which is pathognomonic for cystinosis (23). Cystinosis does not recur in the kidney graft after transplantation has been performed.

## Eye

**Figure 2:**
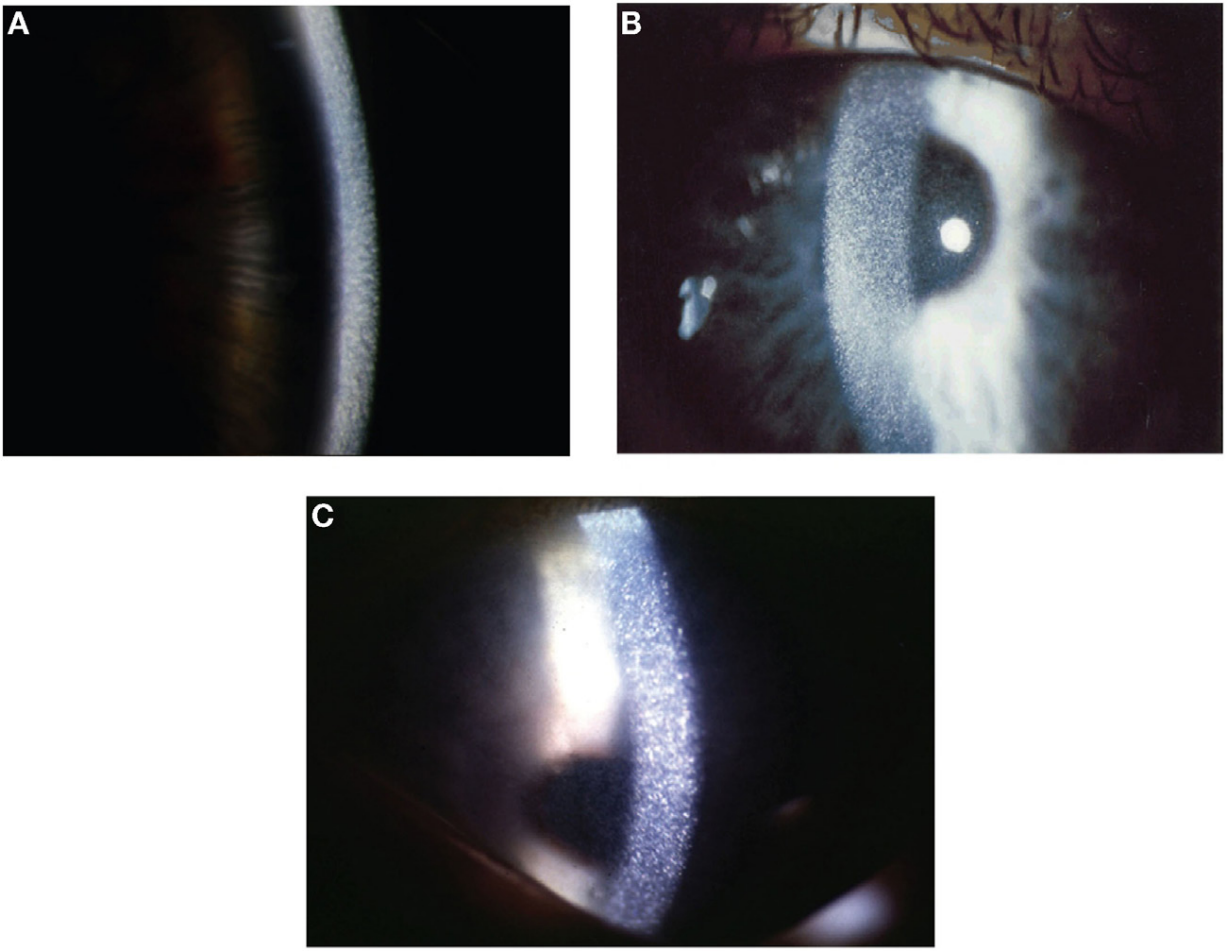
Corneal slit-lamp examination of three patients with nephropathic cystinosis. **(A)** High magnificent slit beam view of cornea with cystine crystals. **(B)** Retroillumination showing marked diffuse crystals packed within the cornea. **(C)** Retroillumination with patient looking down showing the extent of crystal deposition up to the peripheral edge of the cornea. With friendly permission from Clinical Ophtalmology, Dove Press (27).

Probably, the subjectively most impairing early problem is photophobia due to corneal deposition of cystine crystals (see Figure 2) which begins at a median age of 3–4 years, when no treatment is offered. The natural course of cystinosis in the eyes leads to blepharospams, corneal erosions, superficial punctuate keratopathy, and band keratopathy. Involvement of the retina, which can be seen constantly without treatment, causes loss of vision later in life (24, 25). Oral depletion treatment with cysteamine has no influence on the ocular manifestation since there is no vascularization in the cornea. So a topical treatment has been established, bearing new challenges for the patients because cysteamine eye drops have to be applied 6–12 times per day (26).

## Bone and Muscle

Increased urinary loss of phosphate, calcium, and disturbances in vitamin D metabolism cause hypophosphatemic rickets in cystinosis patients. Clinical signs are genua vara, frontal bossing, rachitic rosary, and metaphyseal widening on skeletal X-rays (12). However, there are patients who appear clinically similar without the laboratory findings of disturbed vitamin D metabolism mentioned above despite being treated with cysteamine. This finding might be explained by copper deficiency due to cysteamine toxicity, which may interfere with collagen cross-linking (28). Growth retardation is a frequent finding in cystinosis patients and is usually treated with growth hormone.

Even though X-ray absorptiometry shows bone densities within the normal range, bone fractures can be seen more often in cystinosis. Possibly, it might be that intra-osseous cystine crystals lead to falsely elevated X-ray absorptiometry levels making X-ray absorptiometry an ineffective tool to assess fracture risk in cystinosis patients (29). Three-dimensional peripheral computed tomography should be the preferred method to assess fracture risk in growth-retarded children with (chronic kidney) disease (30).

## Neurology

Accumulation of cystine crystals in the brain leads to neurocognitive impairment. Early signs for brain involvement are nonverbal learning difficulties resulting in poor executive functions, whereas verbal and general intelligence are normal (6, 31). Cysteamine can cross the blood–brain barrier. Depletion treatment with cysteamine can improve neurological outcome even when patients are already symptomatic, but it has also been shown that impairment in visual-motor function occurs even if cysteamine treatment was started before neurologic impairment became noticeable. This suggests that it is not only accumulation, which causes learning difficulties in cystinosis patients (32, 33). Long-term neurological complications are cystine encephalopathy, presenting with cerebellar and pyramidal signs, mental deterioration and pseudo-bulbar palsy, as well as distal myopathy, which begins with weakness of the extremities and results in dysphagia and pulmonary insufficiency. Treatment with cysteamine has been shown to have a beneficial and potential reversible effect on both cystine encephalopathy and distal myopathy (34, 35).

## Endocrinology

The most frequent endocrine finding is hypothyroidism, which appears approximately in half of all cystinosis patients and can easily be treated with substitution of thyroid hormones (6). Another frequent finding is hypogonadism. Male cystinosis patients are prone to be infertile due to azoospermia even if treated with cysteamine since early age. It has been shown that spermatogenesis at testicular level can be intact. The underlying mechanisms remain unclear (36). A successful conception after percutaneous epididymal sperm aspiration followed by intracytoplasmic sperm injection has recently been reported (37). Female cystinosis patients are fertile and several successful pregnancies have been described (38). Other affected endocrine organs are exocrine and endocrine pancreas resulting in diabetes mellitus in 5% of all patients (6, 12).

## Systemic Depletion Therapy

**Figure 3:**
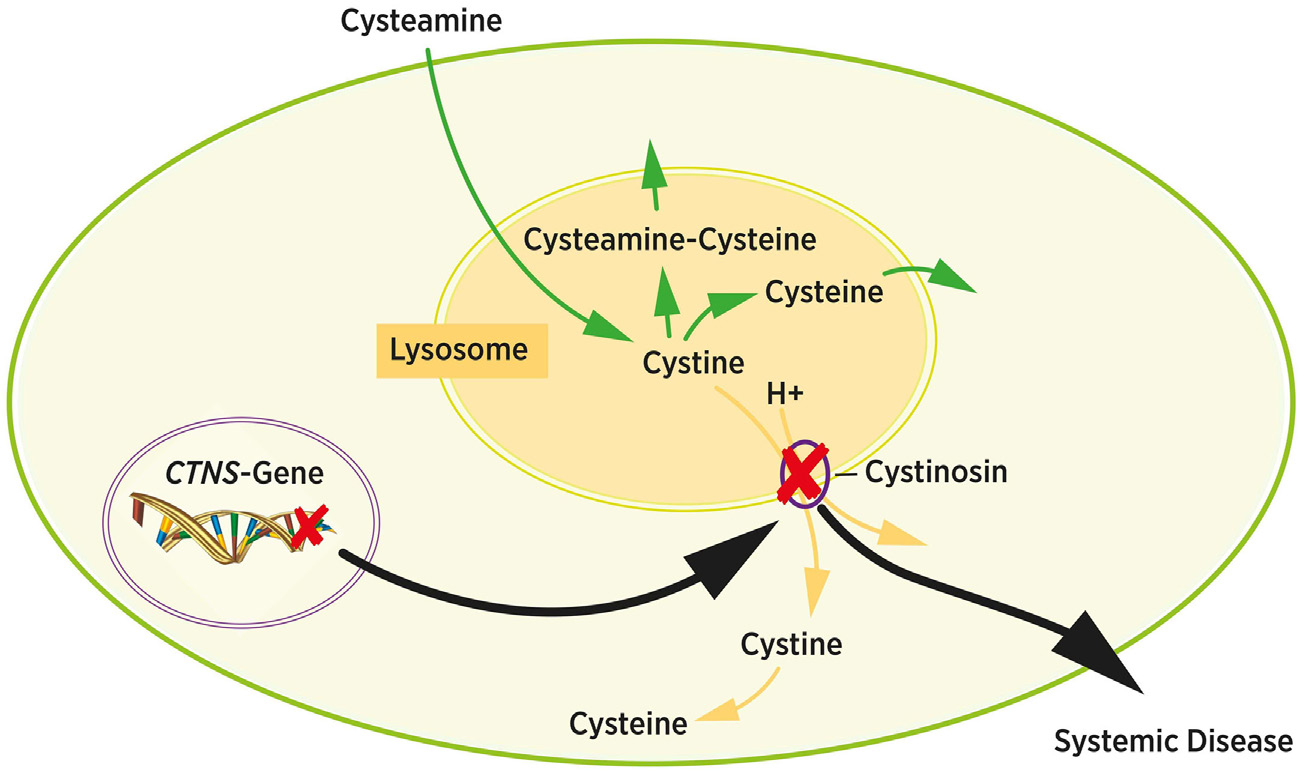
Intracellular cystine metabolism. Cystine normally leaves the lysosome *via* cystinosin by cystine-proton symport (yellow arrows). Mutations in the *CTNS* gene cause cystine accumulation in the lysosome due to dysfunction of cystinosin (black arrows). Cystin accumulates and forms crystals inside the lysosome. Cysteamine induces a chemical reaction resulting in cysteamine–cysteine and cysteine. Both molecules exit the lysosome bypassing the cystinosin transporter protein (green arrows). Adapted from Ref. (6).

The first trials to deplete cystine from cells used 1,4-dithiothreitol (DTT) or ascorbic acid with moderate success (19). The cystine depleting therapy with cysteamine was first described in 1976 and is still the golden standard in cystinosis therapy (6, 39). Cysteamine induces a thiol-disulfide interchange reaction that generates equimolar amounts of cysteine and cysteine-cysteamine molecules from cystine (40). These molecules can exit the lysosomes using alternative cationic transporters and bypass the defective cystinosin transporter, as shown in Figure 3 (41). Cysteamine treatment can be monitored by measuring intracellular cystine levels in white blood cells, which is considered to reflect the cystine concentration of the body’s other tissues. Target levels are usually <1.0 nmol hemicystine/mg protein. Since cystine levels in healthy people are <0.2 nmol hemicystine/mg protein the optimal range for cystinosis patients is considered to be <0.5 nmol hemicystine/mg protein (12, 42, 43).

Cysteamine has been available as a commercial drug since 1997 in an immediate-release formulation that demands a strict 6-h administration schedule to maintain effective plasma levels. A new delayed-release formulation has been available since 2013 in the USA and since 2014 in Europe which must only be administered twice daily. Both formulas use cysteamine bitartrate since the bitartrate formulation needs lower doses to maintain plasma levels than cysteamine hydrochloride or phosphocysteamine (44). The new delayed-release formulation is composed of enteric-coated, microspheronised beads encapsulated in hard gelatin that allow to extend the intake up to 12 h (45). The maximum plasma levels of delayed-release cysteamine are reached about 3 h after administration, whereas maximum levels of the immediate formulation can be found after about 1 h already (46). Both extended- and immediate-release cysteamine have been proven to reach cystine target levels, which are thought necessary to slow down progress of cystinosis-related symptoms. The 12-h administration of the extended-release formulation supports therapy adherence by simplifying the administration schedule whereas experiences of side effects differ from center to center (43, 45, 47). Since new therapeutic strategies in many fields of pediatric diseases enabled patients to reach adolescence and adulthood, e.g., patients with cystic fibrosis or diabetes mellitus, new problems with therapy adherence at this age arose leading to the concept of a controlled transition from pediatric to adult health-care services. This can also be seen in cystinosis patients for whom special transition protocols are recommended (48, 49).

Most reported side effects of cysteamine therapy are halitosis, disagreeable sweat odor, and gastrointestinal side effects like nausea and abdominal pain. Proper dosing up to a maximum of 1.95 g/m^2^/day with a gradually increasing application schedule avoids side effects as lethargy, hyperthermia, and rash (50–52). Recent reports of new adverse events like bruise-like skin lesions, bone abnormalities, and muscle weakness in cystinosis patients with Fanconi syndrome showed that they have an increased urinary copper excretion under cysteamine therapy. This led to the hypothesis that cysteamine toxicity causes copper deficiency because of the structural similarity of cysteamine to D-penicillamine resulting in a reduced formation of aldehydes required for collagen cross-linking (28).

## Eye Drops

Systemic depletion therapy with cysteamine reduces posterior segment complications like pigmentary changes that can lead to retinopathy and loss of vision, but does not prevent deposition of cystine crystals in cornea and conjunctiva. It has been shown that photophobia is associated with crystal density, infiltration of inflammatory cells, and nerve damage within the cornea. Therefore, a topical treatment is necessary and has proven to be effective (24, 53, 54). Cysteamine eye drop formulations are aqueous solutions that have to be administered 6–12 times per day. Because cysteamine is unstable at room temperature and to light exposure, storage and transport of the eye drops are challenging making therapy adherence difficult. A new, gel-like viscous formulation with a fivefold higher concentration of cysteamine has been developed, which has to be administered only four times daily. The gel-like formulation increases the contact time to the cornea allowing the cysteamine to penetrate more deeply into the corneal layers. On the other hand, using the gel-like drops produce side effects like stinging, burning, and vision blurring that were more common compared to the aqueous formulation. The reason may be the higher cysteamine concentration and the viscous consistency but it did not lead to lower therapy adherence (24). Patients untreated with eye drops sometimes develop severe corneal lesions that require a corneal transplant. A topical treatment is necessary even after corneal transplantation because cystinosin-deficient host cells can reinvade into the transplanted cornea (14).

## Future Perspectives

Even though the depleting therapy with cysteamine—oral and topical—has improved the prognosis of cystinosis patients dramatically, it is still not a curative therapy because the defective lysosomal transport protein cystinosin is only bypassed. New formulations of oral and topic cysteamine can alleviate the medical schedule and through this support therapy adherence. But cysteamine has only a delaying effect on complications like end-stage renal failure. Evidence grows that dysfunction of the lysosomal transport protein cystinosin leads to several disturbed intracellular interactions that cannot be corrected by depleting cystine from the lysosomal compartment: for example, cystinotic cells show (i) impaired chaperone-mediated autophagy (55); (ii) reduced levels of transcription factor EB, a key factor in regulating lysosomal biogenesis and clearance (56); and (iii) downregulation of the mammalian target of rapamycin pathway in proximal tubular cells (57). All these mechanisms are not influenced by cysteamine treatment and are associated with the persistence of renal Fanconi syndrome (8). Also altered cellular energy homeostasis or increased oxidative stress found in cystinotic fibroblasts offer new potential targets for the treatment of cystinosis, but still do not offer a causal therapy (58, 59).

The most promising approach to cure cystinosis is the transplantation of hematopoietic *CTNS*-carrying stem cells. Stem cells from wild-type donors were transplanted into irradiated *CTNS*-knockout mice (60). Whereas transplantation of mesenchymal stem cells did not integrate efficiently, the transplantation of hematopoietic stem cells led to stable engraftment in mice. This resulted in a long-term improvement of renal function including Fanconi syndrome even though the stem cells did not reprogram proximal tubular cells. Confocal microscopy showed that most transplanted stem cells differentiated into interstitial lymphoid, dendritic, or fibroblastic cells and did not replace renal epithelium cells (61). The reduction of cystine content in different tissues reached up to 94% (60).

Transplantation of hematopoietic stem cells was still effective in older mice, suggesting that stem cell therapy might still be an option even if tissue injury is already manifested. The exact mechanism of stem cell therapy remains unclear. It is possible that stem cell therapy protects healthy tissue from being harmed by cystine crystals or that damaged tissue is being reversed (8). Further problems of allogeneic stem cell transplantation like graft-versus-host-disease, which cause high morbidity and mortality in transplanted patients, still need to be considered. A new approach uses genetically modified autologous hematopoietic stem cells. For transfer a self-inactivating-lentivirus vector is used in an attempt to lower the risks of allogeneic stem cell transplantation (62).

## Transition and Transfer

The above-mentioned recommendations of structured transition programs (48, 49) must by no means attach little value but transition has paramount importance in rare diseases that require interdisciplinary lifelong care. Pediatric cystinosis patients in general experience comprehensive, interdisciplinary, and structured care led and coordinated by pediatric nephrologists with the Fanconi tubulopathy and chronic kidney disease being the leading symptoms. This coordinated guardian care may change when the patient is transferred to adult care. Lack of knowledge about rare diseases might be a problem just as appointments with separate professionals focusing on their own specialties resulting in “fragmented” care (48). Therefore, in Germany a current initiative aims to transfer pediatric cystinosis patients to adult Morbus Fabry centers. By this, young adult patients encounter a patient-centered care with high awareness of rare diseases that is led by an adult nephrologist and provides interdisciplinary resources and institutional support perfectly applicable to the needs of cystinosis patients.

## Conclusion

Over the last decades, our knowledge and understanding of cystinosis has improved continuously. This led to new therapy options and simplified medicine formulations, which dramatically improved life expectancy and life quality of cystinosis patients. But still there is no curative therapy. The new approach of stem cell transplantation gives hope to become a curative treatment that would mean another big step for cystinosis patients to further improve life expectancy and quality.

## Author Contributions

SB and LW were responsible for concept and creation of this manuscript. Both authors revised the manuscript and approved the final version to be published.

## Conflict of Interest Statement

LW has received travel grants and speaker’s honoraria of Raptor Pharmaceuticals, Horizon Pharma, and Chiesi GmbH. SB has received travel grants and speaker’s honoraria of Orphan Europe declares no conflict of interest.
